# Daily Physical Training Improved Coronal Imbalance of Adult Degenerative Scoliosis: A Case Report

**DOI:** 10.3390/medicina59081443

**Published:** 2023-08-09

**Authors:** Koji Akeda, Takahiro Hasegawa, Koki Kawaguchi, Junichi Yamada, Norihiko Takegami, Tatsuhiko Fujiwara, Akihiro Sudo

**Affiliations:** Department of Orthopaedic Surgery, Mie University Graduate School of Medicine, Tsu 514-8507, Japan; hasegawa-t@med.mie-u.ac.jp (T.H.); k-kawaguchi@med.mie-u.ac.jp (K.K.); yamada-j@med.mie-u.ac.jp (J.Y.); n-takegami@med.mie-u.ac.jp (N.T.); tatsuhiko-f@med.mie-u.ac.jp (T.F.); a-sudou@med.mie-u.ac.jp (A.S.)

**Keywords:** adult spinal deformity, conservative treatment, exercise

## Abstract

*Background and Objectives*: Adult (de novo) degenerative scoliosis (ADS) develops through degenerative changes in the lumbar spine, leading to spinal malalignment, which usually progresses with age. Strong evidence for non-operative care in patients with ADS is lacking, and whether physical exercise can improve the scoliosis curve remains unknown. *Materials and Methods:* We present a case of early stage ADS in which the coronal imbalance was improved by daily training. A 65-year-old female patient complained of lower back pain (LBP) and bilateral leg pain. She was diagnosed with early stage ADS with lumbar degenerative spondylolisthesis by imaging. She completed six months of daily physical training, including swimming, aerobic bikes, stretching, yoga, and Taijiquan. *Results*: Her LBP and neurological symptoms improved, and coronal–spinal balance was restored, which was maintained for four years by continued daily physical training. *Conclusions*: This is the first case of a 65-year-old ADS patient whose coronal balance was significantly restored through daily physical training. Substantial physical training focused on trunk muscle strength is important for spinal stabilization and for improving spinal malalignment in patients with early stage ADS.

## 1. Introduction

Adult (de novo) degenerative scoliosis (ADS) is a condition of the aging population in which a lumbar scoliosis curve develops without preexisting spinal deformity [1]. The causes of ADS are multifactorial and include genetic predisposition and intervertebral disc (IVD) degeneration. ADS develops through asymmetric IVD and facet degeneration, typically in the lumbar spine, which leads to sagittal and/or coronal plane malalignment that usually progresses with age [1,2]. Patients with ADS usually present with progressive lower back pain (LBP) and/or neurological symptoms such as canal stenosis or degenerative spondylolisthesis.

Non-operative treatments, including physical therapy, analgesics, and injection therapies, are regarded as the primary options for managing pain and maintaining physical function; however, there is a lack of strong evidence for this treatment [1,2]. Physical exercises, including spinal stretching and deep trunk muscle training, is considered to improve the mobility and deformity between vertebrae. Recent studies have reported that specific exercises, including strengthening of the back and abdominal muscles and active self-correction, are essential for improving spinal stability and deformity curves in patients with adult idiopathic scoliosis [3,4,5,6,7,8]. However, whether physical exercise can improve the scoliotic curve in patients with ADS remains unknown.

Herein, we present a case of early stage ADS in which coronal imbalance was improved by daily training.

## 2. Case Report

### 2.1. Patinet Information

A 65-year-old female patient presented to our institution with low back pain (LBP) and bilateral leg pain. She had been treated conservatively by pharmacotherapy using non-steroidal anti-inflammatory drugs (NSAIDs) and Limaprost at a local orthopedic clinic for three years. She has been swimming three times a week; however, her symptoms worsened, and she recently experienced difficulty walking. She experienced severe LBP and posterior thigh pain while standing for long periods and during long-distance walking; however, no obvious muscle weakness was observed in either lower extremity. No bladder or rectal disorders were observed.

### 2.2. Image Examinations at First Visit

**Figure 1:**
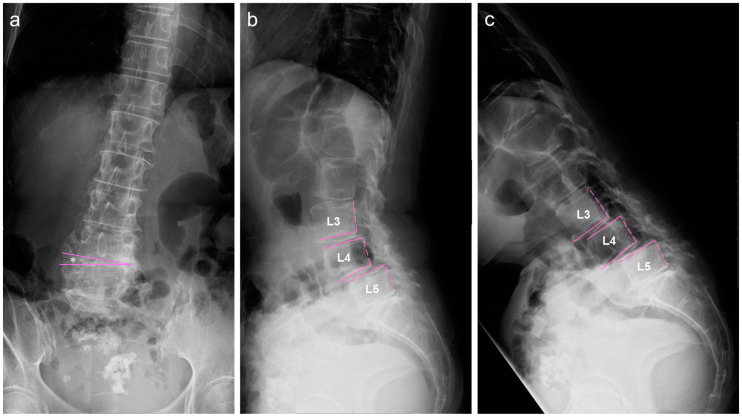
Standing lumbar radiographs at baseline. (**a**) Antero-posterior view, (**b**) lateral neutral position, (**c**) lateral forward-bending position. Straight lines indicate the margin of vertebral endplates, and spotty lines indicate the margin of posterior wall of vertebrae. * indicates 12° of L4 coronal tilt.

**Figure 2:**
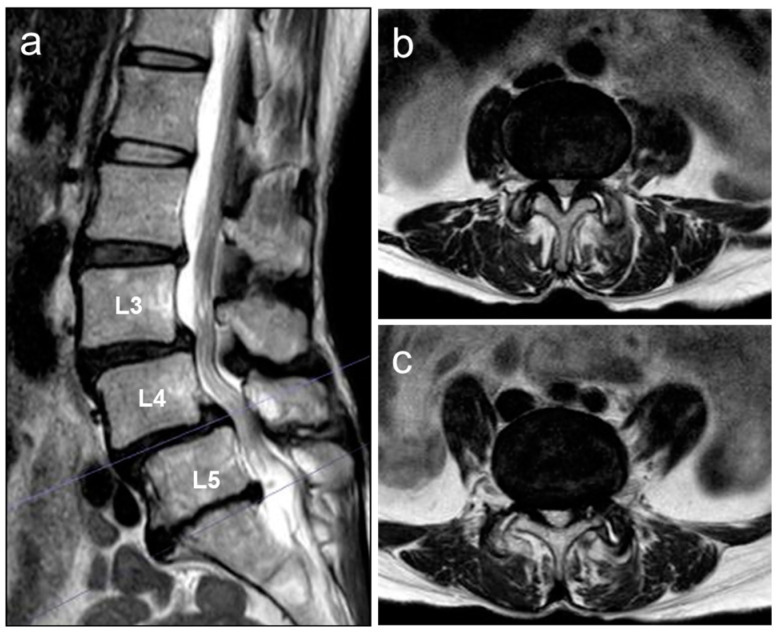
T2-weighted magnetic resonance imaging of the lumbar spine at baseline. (**a**) Midsagittal section, (**b**) axial section at L3/L4, and (**c**) axial section at L4/L5.

A standing lumbar radiograph in the antero-posterior view showed a coronal imbalance of the lumbar spine with L4 coronal tilt (12°) (Figure 1a). Lateral lumbar radiography revealed spondylolistheses at L3 and L4 (Figure 1b). A forward-bending radiograph showed an increase in anterior slipping of the L3 and L4 vertebrae (Figure 1c). A T2-weighted image (T2-WI) of sagittal lumbar magnetic resonance imaging (MRI) demonstrated narrowing of the lumbar canal at the L3/L4 and L4/L5 disc levels (Figure 2a). Axial lumbar MRI (T2-WI) showed definite stenosis (Grade C by Schizas’s grading [9]) narrowed by a disc bulge and thickened ligamentum flavum at the corresponding levels (Figure 2b,c).

**Figure 3:**
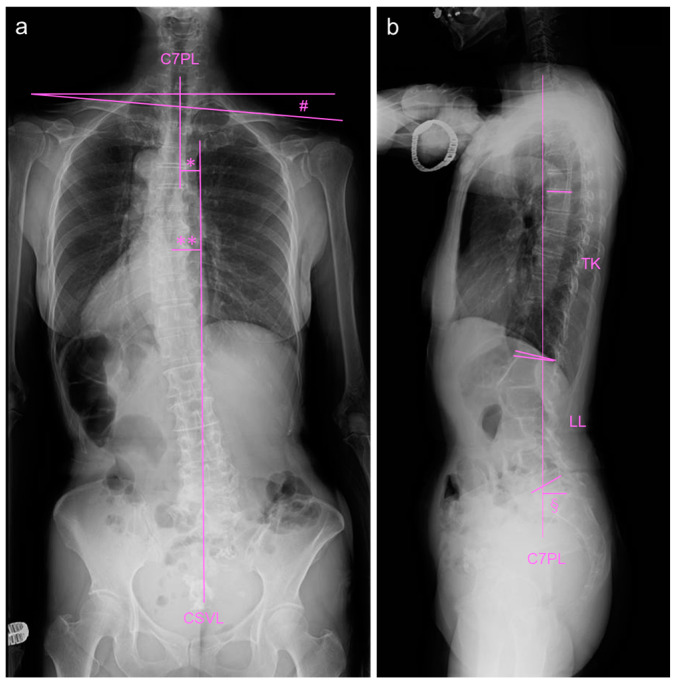
Standing whole-spine radiographs at baseline. (**a**) Antero-posterior whole-spine radiograph. # indicates clavicle angle, * indicates C7-CSVL measurement, and ** indicates apex-CSVL measurement. (**b**) Lateral whole-spine radiograph. C7PL: C7 plum line, TK: thoracic kyphosis, LL: lumbar lordosis, sagittal vertical axis: SVA. § indicates sagittal vertical axis (SVA).

An antero-posterior whole-spine radiograph in standing position showed a coronal imbalance (Figure 3a). The C7 central sacral vertical line (CSVL) measurement was 20 mm (normal approximately 0 mm), the apex (T8/T9) CSVL was 30 mm (normal approximately 0 mm), the Cobb angle of the compensatory thoracic curve was 19°, and the clavicle angle was 4.7° (normal approximately 0°). Lateral whole-spine radiography revealed mild sagittal imbalance, including pelvic parameters (Figure 3b). The sagittal vertical axis (SVA) measurement was 32 mm (normal approximately 0 mm), thoracic kyphosis (TK) (T5–T12) was 12° (normal approximately 20°), and lumbar lordosis (LL) was 41° (normal approximately 50°). The sacral slope (SS) was 28° (normal approximately 35°); pelvic tilt (PT), 27° (normal approximately 15°); pelvic incidence (PI), 54° (normal approximately 50°); PI-LL, 13° (normal approximately 0°). According to the SRS-Schwab classification [10], the curve is type T, PI-LL: +, SVA: 0, and PT: +.

### 2.3. Daily Physical Training

**Figure 4:**
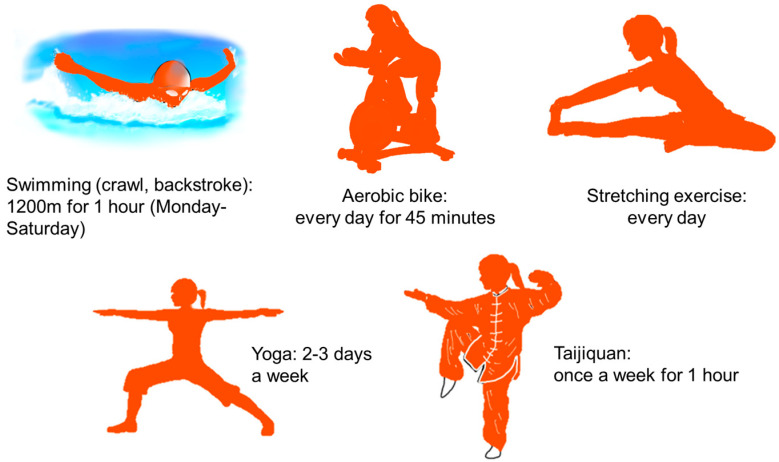
Daily physical exercises in a week.

The patient completed the following daily physical exercises focusing on training the deep trunk muscles and stretching the lumbar ligaments (Figure 4). (1) Swimming (crawl and backstroke) 1200 m (M) from Monday to Saturday; (2) aerobic bike: 45 min every day; (3) stretching exercise: everyday; (4) yoga: 2–3 days a week; (5) Taijiquan; once a week for one hour.

### 2.4. Image Examinations after Six Months of Daily Physical Exercise

**Figure 5:**
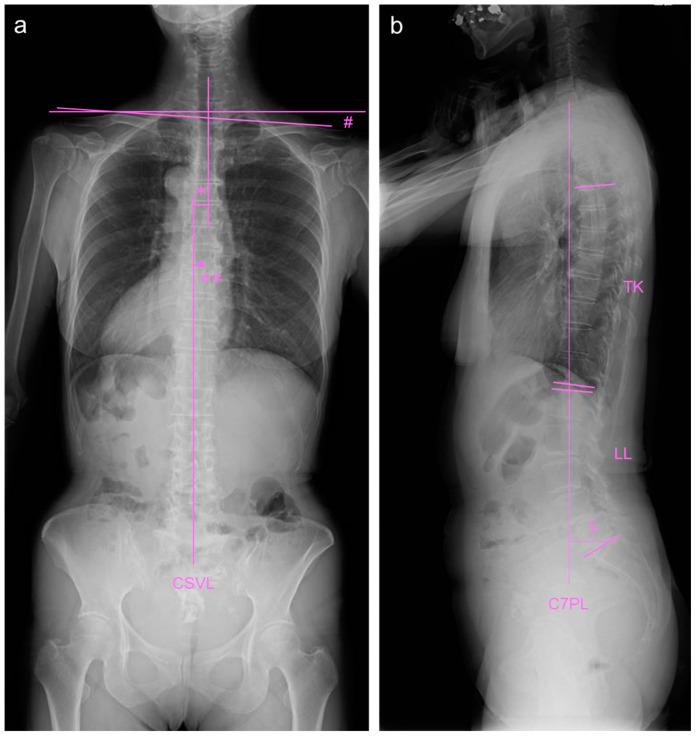
Standing whole-spine radiographs after six months daily physical exercises. (**a**) Antero-posterior whole-spine radiograph. # indicates clavicle angle, * indicates C7-CSVL measurement, and ** indicates apex-CSVL measurement. (**b**) Lateral whole-spine radiograph. C7PL: C7 plum line, TK: thoracic kyphosis, LL: lumbar lordosis, sagittal vertical axis: SVA. § indicates sagittal vertical axis (SVA).

Her LBP and leg pain ameliorated after six months of daily physical exercise. An antero-posterior whole-spine radiograph in standing position revealed an improved coronal imbalance (Figure 5a). C7-CSVL measurement was reduced to 11 mm (from 20 mm at the first visit, normal approximately 0 mm); apex (T8)-CSVL was −5.0 mm (from 30 mm, normal approximately 0 mm); Cobb angle of compensatory thoracic curve was 7° (from 19°); clavicle angle was 2.0° (from 4.7°, normal approximately 0 mm). Lateral whole-spine radiography showed no remarkable changes, with mild sagittal imbalance, including changes in pelvic parameters (Figure 5b). The sagittal vertical axis (SVA) measurement was 45 mm (normal approximately 0 mm), thoracic kyphosis (TK) (T5–T12) was reduced to 12° (normal approximately 20°), and lumbar lordosis (LL) was 34° (normal approximately 50°). The sacral slope (SS) was 20° (normal approximately 35°); pelvic tilt (PT), 32° (normal approximately 15°); pelvic incidence (PI), 53° (normal approximately 50°); PI-LL, 19° (normal 0°). According to the SRS-Schwab classification [10], the curve is type T, PI-LL: +, SVA: 0, and PT: +.

**Figure 6:**
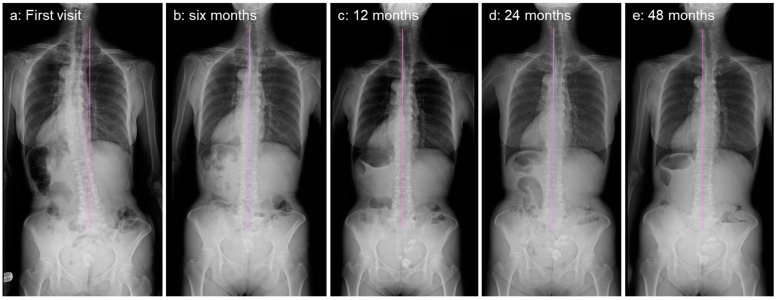
Standing antero-posterior whole-spine radiographs at first visit (**a**), 6 months (**b**), 12 months (**c**), 24 months (**d**), and 48 months (**e**). Straight vertical line indicates CSVL.

**Figure 7:**
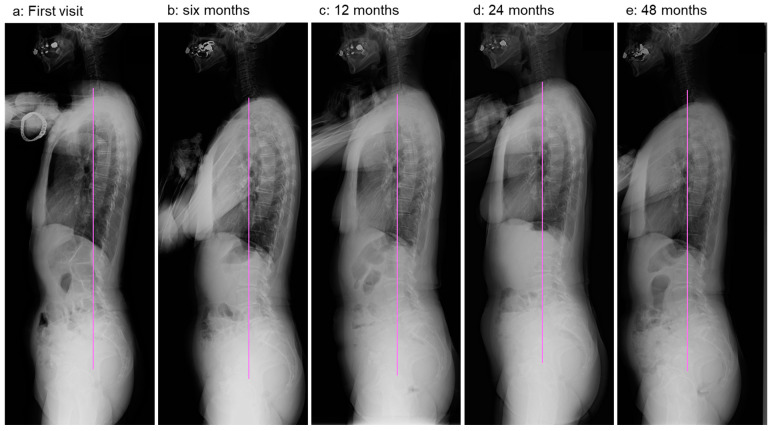
Standing lateral whole-spine radiographs at first visit (**a**), 6 months (**b**), 12 months (**c**), 24 months (**d**), and 48 months (**e**). Straight vertical line indicates C7PL.

Thereafter, she continued daily physical training for four years. Her LBP and LBP-related QOL improved four years after the first visit; however, her leg pain persisted. Her standing whole-spine radiographs showed no worsening of coronal balance (Figure 6) or significant changes in sagittal balance after four years of observation (Figure 7).

## 3. Discussion

The pathophysiology of ADS involves changes in the structure and function of the spine due to age-related degeneration. The degenerative process of ADS is initiated by asymmetric intervertebral disc degeneration, which leads to pathological changes in load-bearing at multi-level IVD and facet joints, resulting in bone remodeling and instability at these changes [1,2]. These changes further lead to progressive fragility of the spinal ligaments and paraspinal and truncal muscles, ultimately leading to a dynamic pattern of curve progression and three-dimensional deformity [1,2,11]. Generally, the scoliosis curve of the patients with ADS progresses with age [12,13,14].

In our case, a standing lumbar radiograph showed a wedge of the L4/L5 disc with degenerative spondylolisthesis. Standing whole-spine radiography showed a mild coronal imbalance with 12° L4 vertebral tilting and normal sagittal global alignment, suggesting that the patient presented with early stage degenerative scoliosis at the first visit. Contrary to previous reports [13], the patient’s coronal imbalance improved after six months of daily physical exercise.

Non-operative treatment is generally conducted as the first-line treatment, although there is no strong evidence for treatment methods [1,2]. Teles et al. [15] conducted a systematic review of the effectiveness of operative and non-operative treatments for adult spinal deformities and reported that non-operative care did not demonstrate significant changes in quality of life during a year’s follow-up. Everett et al. [16] also reported in a systematic review that physical therapy and chiropractic care provided level IV evidence in the management of adult scoliosis.

On the other hand, scoliosis-specific exercises with other types of physical treatment have been proposed to reduce or avoid curve progression by the International Scientific Society on Scoliosis Orthopaedic and Rehabilitation Treatment (SOSORT) [17]. The Scientific Exercise Approach to Scoliosis (SEAS) is based on a specific active correction technique performed without external aid and incorporated into functional exercises [8]. Recently, Negrini et al. [5] reported that SEAS was effective in obtaining curve stability and, in some cases, reducing the Cobb angle in patients with adult idiopathic scoliosis. The authors speculated that the reduction in the scoliotic curve by SEAS could be attributed to the activity of specific muscles and consequent postural collapse.

The current patient, for the first time, showed significant improvement in coronal balance through daily exercises, including swimming, aerobic cycling, stretching, yoga, and Taijiquan. It remains unclear which exercise is most effective; however, these exercises would strengthen the intrinsic spinal muscles, including the erector spinae and multifidus. Moreover, stretching, yoga, or Taijiquan may stretch the concave lumbar ligament and adjust trunk balance. 

Core stabilization (CS) exercise therapy involves training the deep trunk muscles by controlling the trunk in static postures and functional activities, which can activate the transversus abdominis and lumbar multifidus [18]. Recently, it has been reported that CS exercise plays a role in improving curve magnitude, trunk deformity, and pain intensity in idiopathic scoliosis [3,19], suggesting that training of the deep truck muscle is essential for inhibiting curve progression. Although the effect of the CS exercise in patients with ADS remains unknown, it may have a similar effect on curve improvement in these patients.

The current case also showed that the coronal and sagittal balance remained unchanged during the four years of daily training. Furthermore, neither the extent of spondylolisthesis nor the neurological symptoms deteriorated during follow-up. Faraj et al. [20] conducted a systematic review study to identify the prognostic factors for curve progression in de novo degenerative lumbar scoliosis and reported that increased disc degeneration and apical vertebral translation ≥6 mm are associated with curve progression. Interestingly, they also found that degenerative spondylolisthesis was not a risk factor. In the current case, the radiological risk factors for curve progression reported by Faraj et al. [20] were not identified six months after the first visit, and neither the coronal nor sagittal imbalances worsened during the four years of daily physical training.

However, she was in good health, both physically and mentally, and was able to perform hard exercises every day; this case may be a small fraction among women of the same age. Nonetheless, the course of the current case suggests the possibility that adequate sufficient daily physical training may improve the curve progression of ADS, especially at an early stage, and maintain spinal balance for four years.

## 4. Conclusions

We report the first case of a 65-year-old ADS patient whose coronal balance significantly improved through daily physical training. Substantial physical training focused on trunk muscle strength and stretching the lumbar ligaments is important for spinal stabilization and the restoration of spinal malalignment in patients with early stage ADS. It is essential to start with light exercise and gradually increase the exercise intensity and duration according to the individual’s health condition and exercise capacity.

## Data Availability

Not applicable.
